# Insect Decline in the Anthropocene: Historical Parallels and Emerging Monitoring Tools

**DOI:** 10.3390/insects16080841

**Published:** 2025-08-15

**Authors:** Dani Sukkar, Jairo Falla-Angel, Philippe Laval-Gilly

**Affiliations:** 1 Université de Lorraine, INRAE, LSE, F-54000 Nancy, France; jairo.falla-angel@univ-lorraine.fr (J.F.-A.); philippe.laval-gilly@univ-lorraine.fr (P.L.-G.); 2 Plateforme de Recherche, Transfert de Technologie et Innovation (PRTI), IUT Thionville-Yutz, Université de Lorraine, 57970 Yutz, France

**Keywords:** ecosystem monitoring, atmospheric oxygen, historical ecology, entomological conservation, biotic stressors, resilience modeling, AI-based biodiversity tools, climate-driven selection

## Abstract

Insects, the most diverse group of animals on Earth, have historically evolved under strong environmental selective pressures, particularly fluctuations in atmospheric oxygen and temperature. During the Anthropocene, rapid climate change, pollution, and habitat alteration now impose new and compounded stresses, accelerating insect decline at unprecedented rates. Here, we present a conceptual framework comparing ancient environmental drivers of insect physiology, size, and diversity with modern anthropogenic stressors. This perspective reveals how contemporary pressures such as pesticide-induced hypoxia, climate-driven size alterations, and habitat fragmentation resemble and intensify ancient evolutionary constraints. We further highlight the disruption of key ecological services and the emergence of novel biotic pressures, including intensified competition and predation. Recent advances in trait-based modeling, environmental DNA analysis, remote sensing, and AI-powered monitoring offer promising avenues for assessing these complex interactions. Integrating these modern tools with historical evolutionary insights is essential for improving risk assessments, informing conservation strategies, and mitigating the cascading effects of insect diversity loss on ecosystems.

## Simple Summary

Insects are crucial to the environment, supporting ecosystems by pollinating plants, breaking down waste, and controlling pests. However, insect populations are rapidly declining due to human activities like pesticide use, habitat destruction, and climate change. This decline threatens biodiversity and food security. Interestingly, many of today’s anthropogenic pressures resemble natural environmental changes that insects faced millions of years ago, such as shifts in oxygen levels and global temperatures that once influenced their body sizes and diversity. By comparing past and present conditions, we can better understand the present insect decline and predict future trends. Modern tools like DNA-based monitoring, satellite imaging, and artificial intelligence can help us track insect populations more accurately and identify those most at risk. Understanding how modern stressors mirror ancient environmental pressures can improve how we assess threats to insects and help guide better conservation strategies.

## 1. Introduction

Insects are the most diverse and ecologically critical organisms, representing approximately 80% of all animal species [1]. However, insects are experiencing a rapid and alarming decline, a phenomenon often referred to as the “insect apocalypse” [2]. Recent estimates suggest that insect populations are declining up to eight times faster than other animal groups [3], threatening essential ecosystem services such as pollination, nutrient cycling, and pest control. This accelerating loss has raised concerns about profound ecological disruption and the potential triggering of cascading effects across entire ecosystems.

Modernization and the evolution of human civilization have transformed the Earth and atmosphere, rendering it inconvenient for biodiversity and the continuation of many species. Indeed, anthropogenic activity has led to the onset of what is viewed as the sixth great mass extinction event in the age of human advancement, the “Anthropocene” [4]. “The Anthropocene” is a widely used term in environmental sciences to describe the epoch in which human activity has significantly altered Earth’s ecosystems. However, it is important to note that the Anthropocene is not formally recognized as a geological epoch by the International Commission on Stratigraphy (ICS), and its starting point remains debated among scholars from early agriculture to industrialization and the mid-20th century “Great Acceleration.” In this paper, we adopt a conceptual rather than geological use of the term, referring to the onset of large-scale anthropogenic impacts roughly 10,000 years ago to tackle insect population dynamics and evolutionary comparatives.

Insect decline is concerning since insects are integrated into the environmental evolutionary web, and many natural processes are dependent and co-dependent on insects [5]. The ecological services provided by insects range from pollination of most of the world’s crops (80%) maintaining agriculture and plant biodiversity [6,7] to bioremediation of pollutants and facilitating the decomposition of organic material, rendering them crucial for ecological functions [8,9]. According to [10], the decline of insects has received relatively little attention, largely because research priorities have shifted toward larger animals, even though insects dominate terrestrial ecosystems in abundance and ecological function and are declining at a pace exceeding that of many vertebrates [11,12]. The global concern over insect decline was initially driven by widespread reports of honey bee colony collapses, particularly in intensively farmed regions such as the United States, Canada, and Western Europe between 2007 and 2010. These early warning signals served as the catalyst for broader investigations into insect declines across taxa [13,14].

Many studies attribute insect decline, which reduces ecological services and environmental stability, to pollution caused by pesticide application [15,16], with particular concern over neonicotinoids that have prolonged effects on non-target insects, including bees, butterflies, and even insects in aquatic ecosystems [10,17,18,19,20]. Furthermore, the decline in pollinating insect populations threatens global food security and agricultural economies. Anthropogenic activity can destabilize the environment in many ways either directly or indirectly. For example, applying insecticides and fungicides can result in algal blooms, increasing periphyton biomass, and altering invertebrate abundance in aquatic ecosystems [21]. The focus of research on insect decline follows the use of pesticides, agricultural practices, deforestation habitat destruction, and climate change [4]. However, some parameters are often overlooked or not integrated into the study of insect losses.

A compelling observation is that environmental changes during ancient epochs affected insect populations in ways that parallel the impacts of modern anthropogenic activities. Recognizing these parallels offers a valuable perspective: contemporary declines in insect abundance and diversity mirror the consequences of historical environmental shifts. Drawing such comparisons can deepen our understanding of rapid insect decline beyond the widely recognized effects of contamination. Key factors include changes in atmospheric gas composition, notably oxygen and carbon dioxide levels, which have historically and presently influenced insect physiology, body size, and population dynamics [22,23]. Habitat loss and fragmentation, driven today by exponential human population growth, further restrict available space for insects, echoing past periods of environmental constraint. Additionally, global warming and chemical pollution, particularly pesticide use, create stressors that replicate ancient selective pressures, compounding the challenges faced by modern insect species. Therefore, more robust data and insect decline analysis were required for a more appropriate evaluation [24]. Inappropriate risk assessment practices are also implicated in biodiversity decline [16] as they dictate the view on the measures needed to limit insect decline. The International Union for Conservation of Nature (IUCN) Red List only lists 0.9% of known insects, with 26% of them being data-deficient [25], further demonstrating the knowledge gap in insect decline assessments. The effects of weather changes have been linked to increases and decreases in insect biomass, with climate change further threatening insect populations despite occasionally observed conditional increases in their numbers, as explained by [26].

In this article, we explore the largely overlooked connection between anthropogenic activities and their indirect similarity to ancient environmental pressures, and how these processes drive insect decline and evolutionary selection. We highlight how integrating this perspective into environmental research and risk assessment frameworks can enhance our understanding of insect responses to modern stressors. Additionally, we examine key environmental factors that have historically shaped insect evolution, emphasizing how contemporary human activities replicate and intensify these selective forces.

## 2. Insect Species Status

Insects represent approximately 80% of all described animal species, yet they account for only 13.5% of the animals assessed in the IUCN Red List. This stark discrepancy reveals a significant taxonomic and conservation bias, where insect species, despite their numerical dominance, are grossly underrepresented in global conservation assessments. This underrepresentation reflects both the limited funding and attention historically devoted to invertebrate monitoring and the challenges of taxonomic identification across highly diverse insect groups. Moreover, among those insects that have been assessed, a considerable proportion fall into threatened categories, suggesting that existing evaluations may disproportionately target already vulnerable or charismatic species, thus compounding the data gap for less-studied groups.

**Figure 1:**
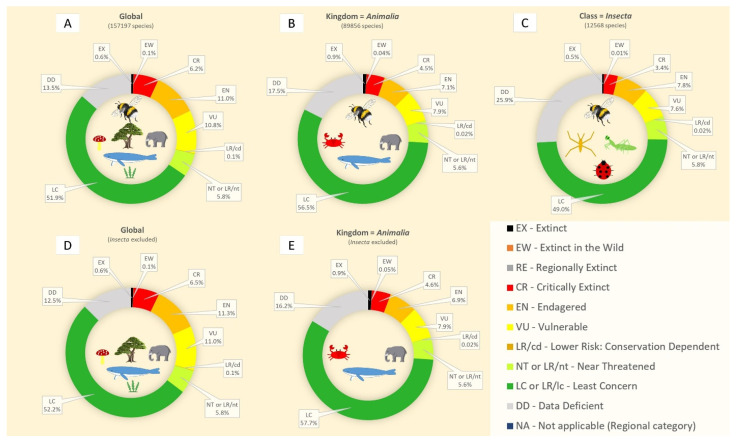
IUCN Red List pie charts representing threatened species categories. The distribution of percentages includes all species (**A**), the kingdom Animalia (**B**), the class Insecta (**C**), all species excluding insects (**D**), and all animals excluding insects (**E**). Data were obtained from the IUCN Red List website until 28 February 2024.

An analysis of the IUCN Red List data (Figure 1) offers further insight. Globally, 51.9% of all listed species are categorized as least concern (Figure 1A). Within the kingdom Animalia, this figure rises to 56.5% (Figure 1B), reflecting the relatively greater conservation stability of many vertebrate groups. However, when focusing solely on insects, the proportion of species listed as least concern drops by 7.5%, reaching only 49% (Figure 1C). This suggests that among the insects that are assessed, relatively more are considered to be at risk compared to the broader animal kingdom. Insects make up only 7.99% of all species and 13.49% of all animals assessed in the Red List, yet their inclusion skews global threat patterns downward. When insects are excluded from the global dataset, the proportion of species considered of least concern increases by 0.3% (Figure 1D), and by 1.2% when excluded from the Animalia kingdom (Figure 1E). These modest but meaningful shifts underscore the contribution of insect data to the overall elevation of perceived extinction risk in biodiversity assessments.

Together, these statistics reinforce that insects, despite their diversity and ecological importance, remain poorly understood in terms of conservation status. This gap likely results in an underestimation of true extinction risk, especially given that many assessed species are already known to be declining or threatened. Furthermore, the large proportion of data-deficient insect species highlights the urgent need for expanded taxonomic and ecological research, as well as improved integration of insects into global conservation frameworks. Addressing this gap is essential not only for understanding the trajectory of insect populations but also for preserving the critical ecosystem functions they support, from pollination and decomposition to nutrient cycling and food web stability. The observation that excluding insects slightly increases the overall proportion of species considered at lower risk further highlights the lack of data to truly assess any underlying disproportionate vulnerability. Additionally, the high percentage of data-deficient insect species may suggest that the true extent of insect decline may be significantly underestimated, reinforcing the urgent need for more focused conservation efforts and comprehensive risk assessments.

The following sections explore the main anthropogenic stressors implicated in insect decline, including habitat destruction, pesticide use, urbanization, and climate change. By comparing these contemporary drivers with historical environmental shifts that influenced insect evolution, we aim to identify recurring patterns and physiological responses that may help predict future risks and conservation strategies.

## 3. Historical Drivers of Insect Evolution: Interactions and Ecosystem Roles

Insect diversity has undergone dynamic changes throughout the Phanerozoic, shaped by a combination of extinction events, environmental shifts, and co-evolutionary processes. Major diversification events occurred during the Carboniferous, Permian, and Cretaceous periods, driven by habitat expansion and the emergence of new ecological niches [27,28,29]. These radiations were not uniform; for example, while some Carboniferous insects, such as Meganisoptera, reached remarkable sizes, most taxa remained moderate in body size, and the fossil record is biased toward larger specimens [30,31]. Insect evolution was also tightly linked with the diversification of terrestrial plants, particularly the rise of angiosperms during the Cretaceous, which gave rise to specialized herbivory and pollination strategies [32]. Additionally, broader ecosystem changes such as the Mesozoic Lacustrine Revolution [33], the spread of grassland biomes [34,35], and the emergence of mammals [36] created new selective pressures and opportunities. Insects were not merely passive responders to environmental change; groups like termites and ants actively shaped ecosystems through bioturbation and biomass accumulation, contributing significantly to biogeochemical cycles [37]. These historical dynamics offer critical insight into the long-term resilience and adaptability of insects and provide valuable context for interpreting current biodiversity loss.

### 3.1. Atmospheric Oxygen and Insect Evolution

From the beginning of life, the highest atmospheric oxygen peak occurred within the Late Devonian and the Early Carboniferous periods, reaching 30–35% [38]. In the Carboniferous period (359–299 mya), the arthropods flourished, particularly insects reaching unprecedented sizes, notably the giant dragonfly relatives of the orders Meganisoptera (formerly Protodonata) and Palaeodictyopteroidea [39]. However, although the Carboniferous produced some of the largest known insect taxa, these were likely exceptional. Most insects remained moderate in size, and fossilization inherently favors larger bodies [40,41]. Nevertheless, high atmospheric oxygen during certain intervals may have temporarily released physiological constraints on maximum body size, providing a useful evolutionary analogue for understanding modern metabolic stressors.

Insect body sizes are related to the concentration of atmospheric oxygen partial pressure (pO_2_), particularly due to the involvement of an adapted tracheal system that benefits from relatively high oxygen levels [22]. Thus, it is valid to state that lower atmospheric oxygen may be implicated in the development of smaller insect body sizes. In addition, the density of atmospheric gas is as important as its concentration as reviewed by [42] elaborating that the cooling event in the Ordovician period (465–455 mya) occurred when CO_2_ was 8–20 times higher than present days while the atmospheric density was as low as 0.6 bar.

It is also imperative that researchers investigate the variation in body sizes within the same species in addition to the body sizes of the present species and elucidate favoritism among species depending on the sizes and complexity of their tracheal system. In addition, along with the presence of insect gigantism, rapid diversification of blattoid insect fauna occurred at the end of the Moscovian (303–307 mya) and during the Kasimovian (307–303 mya) ages within the late Carboniferous period [43], indicating a potential correlation between oxygen levels and insect diversity. In the Early Cretaceous period, the association between insect body size and atmospheric oxygen levels was decoupled, probably due to the coinciding diversification of birds and their advanced maneuverability, which facilitated their dominance and competition with large insects, rendering birds a biotic factor that influenced insect size but not negating the effect of atmospheric temperature on its own [44,45].

### 3.2. Temperature Stress and Insect Body Size Shifts

The larger the insects, the more they display sensitivity to increases in temperature [43]. Thus, the lower temperature in the late Carboniferous period is probably a contributing factor to insect gigantism. The stress induced by warm temperatures is observed to reduce worker production and reduce longevity in adults, in addition to decreasing development time and mean body size, in *Temnothorax nylanderi* (Foerster, 1850) ants [46].

It was found that lower temperatures usually result in a decreased development rate and an increase in both the duration of developmental stages and adult longevity of the lace bug *Corythucha ciliata* Say, 1832 in laboratory conditions [47]. Similar results on the effect of temperature were found with the Lepidopteran *Spodoptera frugiperda* (J.E. Smith, 1797) but with high survivability at higher temperatures [48]. In contrast, a lower survival rate was associated with the Dipteran pumpkin fruit fly *Bactrocera tau* Walker, 1849 when exposed to short-term increased temperatures [49]. Smaller body sizes are also seen to be associated with increasing temperature across the Chironomidae family with a species-specific decrease in wingspan [50]. In addition, the Asian honey bee (*Apis cerana* Fabricius, 1793) showed higher heat tolerance compared to the European honey bee (*A. mellifera* Linnaeus, 1758) due to the smaller body size of the former [51]. Thus, temperature can indeed induce a selective pressure that favors insects of relatively smaller sizes. However, the different heat responses between insect orders and within them must also be considered.

Though the concept of size plasticity in insects is observed, the hereditary genetic basis of size is dominant according to phylogenetic studies in tropical insects [52,53] as an exception to the temperature-size rule [54]. The effect of temperature on insect diversity, body size, and development, in addition to the species-dependent effects, demands a more thorough investigation into the mechanisms underlying insect decline in the context of climate change. Increasing temperature can have a pronounced effect on insects, as seen in a 45-year monitoring of beetle incidence up until the 2010s in New Hampshire (United States) that showed an 83% decrease with warmer temperatures and a reduction in snowpack [55]. The disfavor of some insect species in the context of high temperature needs to be studied more thoroughly, considering the relationship between insect size, species, order, and temperature changes.

## 4. Anthropogenic Stressors Resembling Ancient Pressures

Beyond well-recognized abiotic drivers such as oxygen availability and temperature, modern anthropogenic stressors, particularly chemical pollutants like pesticides, also impose strong selective pressures on insect physiology and evolution. While the mechanisms differ, these contemporary stressors can lead to physiological challenges that are functionally analogous to those encountered during past environmental upheavals. This section explores how anthropogenic activities, including the direct effects of pesticides and the indirect consequences of climate change, constrain insect tolerance to both biotic and abiotic conditions, shaping evolutionary trajectories in ways that echo historical patterns.

### 4.1. Pesticide Exposure and Hypoxia Responses

Low-oxygen hormesis is an example of how anoxia or hypoxia induces a protective response in insects [56]. A protective response was observed when hemocytes from honeybee fifth instar larvae were exposed to pesticides and their synergism [57]. This is intriguing because pesticides have indeed been affiliated with alterations in cellular respiration [21]. Although tracheal respiration and mitochondrial inhibition operate through distinct physiological mechanisms [58], both can constrain metabolic performance and thus act as strong selective filters.

Modern systemic insecticides, such as neonicotinoids, exhibit neurotoxic action at doses several orders of magnitude lower than legacy pesticides like DDT. Their persistence in plant tissues and soils, combined with chronic sublethal effects, have been shown to weaken bee colonies and reduce floral resource availability, creating a feedback loop of nutritional and immunological stress [20]. In fact, systemic pesticides like imidacloprid and pyraclostrobin impair mitochondrial respiration across multiple insect taxa including honey bees, bumblebees, bollworms, and fruit flies, leading to hypoxia-like stress responses [59,60,61,62,63,64,65].

Moreover, the ability to induce hormesis is limited to the tolerance to low oxygen levels. This was observed in *Drosophila melanogaster* (Meigen, 1830), where older specimens were less tolerant to anoxia conditions [66]. In addition, the protective response correlates negatively with immune responses such as phagocytosis in the presence of pesticide mixtures [57]. Henceforth, the advantages of hormesis may be accompanied by increased susceptibility to diseases and infections, which could also contribute to rapid insect decline worldwide. This could explain why some insects become more prone to diseases when exposed to pesticides [67,68,69,70,71]. The cumulative effect of respiratory inhibition and immune suppression diminishes insect resilience, especially under compounded environmental stressors such as elevated temperature and poor nutrition.

### 4.2. Temperature Changes and Insect Immunity

Increasing global temperatures are a major concern in environmental studies due to their impact on the biosphere. In response to rapid temperature changes, organisms such as insects possess heat shock proteins that help maintain proper protein folding under stress conditions, ultimately promoting cell survival [72]. In insects, heat shocks were found to differentially alter gut microbiota and immune effectors [73]. This creates an advantage for some species over others, dysregulating the balance environmentally either in terms of ecological function or competition and predation. Temperature and pathogens exert selective pressure on insects [74]. This insinuates that the immune response and temperature variations go hand in hand in, dictating the ability of insects to defend against pathogens.

Heat shocks were also reported to enhance the immune response in insects via heat-shock proteins that play a role in protection from ROS-induced damage [74]. Temperature-related immune alterations, including cytokine responses such as SRP and GBP, are described in Section 4.2. These responses influence fitness, feeding, and development across several species. The SRP peptide was first identified in the common cutworm *Spodoptera litura* (Fabricius, 1775) and was expressed in response to parasitization by the parasitoid wasp *Microplitis manilae* (Ashmead, 1904), resulting in reduced food consumption, reduced bodyweight, and increased plasmatocyte spreading and attachment [75]. Growth-blocking protein (GBP) and SRP are two cytokines elevated by heat shock exposure in armyworm (*Mythimna separata*, Walker, 1865) larvae, resulting in decreased feeding activity and a reduction in size development with GBP in upstream signaling relative to SRP [76]. This also explains how high temperatures alter insect size development, as mentioned in previous sections. Temperature may also affect the concentration of insect immune cells (hemocytes; haemocytes). Heat acclimation, for example, resulted in decreased hemocyte concentration in the ladybug species *Harmonia axyridis* (Pallas, 1773) with lowered starvation tolerance [77].

The evolutionary direction of insects is thus put into question. With increasing global temperatures and the fact that organisms tend to conserve energy, it is possible that insects may adapt to be less responsive to increased heat, and, thus, heat shocks would not be as inductive. Though this may indicate that insects could adapt to the negative effects of increasing temperatures and further studies are required on the evolutionary effects of temperature change, the development of such species would appear after the toll of the current climate change has already affected most insect species in terms of abundance and diversity—keeping in mind that certain adaptations can occur in response to climate change, as previously discussed concerning insects of prehistoric eras.

## 5. Space Limitations, Light Pollution, and Population Bottlenecks

Sensibly, reproduction increases the number of individuals of a species that take up space. The low availability of space or breeding grounds can be caused as the ultimate result of rapid reproduction, as seen with humans, or external factors such as certain environmental events or the occupation of space by other species could cause it. In addition, the modification of properties in ecological niches can also be considered a modification of space availability for insect expansion and reproduction since variations in these niches may result in the inability or disfavor of insect reproduction. Urbanization, intense agricultural practices, and habitat loss, in addition to other factors, are considered drivers of insect decline worldwide [78].

In addition to habitat loss, pesticide use, and climate change, other important but sometimes overlooked contributors to insect decline include light pollution, biological invasions, and the spread of pathogens. Artificial night lighting disrupts circadian rhythms, foraging behavior, and reproduction in nocturnal insects [79], while invasive species and novel pathogens contribute to competitive displacement and immunological stress.

Beyond these localized drivers, evidence increasingly shows that stressors such as long-range transport of pollutants, atmospheric nitrogen deposition altering plant communities and nutrient cycling [80], and microclimate disruptions driven by climate change [81] also contribute to insect decline even in protected or montane environments. Additionally, phenological mismatches between insects and their floral or prey resources due to warming have been observed in high-elevation or “pristine” habitats [82]. These findings underscore the systemic and multifactorial nature of global insect decline and caution against assuming protected status is sufficient to buffer populations from anthropogenic pressures.

In the case of limited areas, evolution and natural selection usually direct organisms to either decrease their reproduction rate and/or smaller sizes that occupy less space. In the case of insects, deforestation and urbanization are already stripping away natural habitats, thus limiting the available surfaces where insects can thrive and reproduce. To add, the nutritive state affects insect development and controls body size via nutrient-dependent regulation [23,83]. With the high demand for nutrition not being met, survivability will favor insects that do not consume as much, resulting in fewer relatively larger insects and ultimately in decreased insect diversity. This takes into consideration the life cycles of insects and their durations, which have variable nutritive and environmental requirements.

## 6. Climate Change Shifts Scales Between Pest and Beneficial Insects

### 6.1. Impacts on Parasitoids Wasps and Pest Insects

In insects, temperature affects the success of parasitic infections. The parasitoid wasp *Leptopilina boulardi* Barbotin, Carton & Keiner-Pillault, 1979 has a higher parasitic success rate in *D. melanogaster* and *D. yakuba,* Burla, 1954 with increasing temperature [84]. The mechanism involves altering the immune interaction between host and parasitoid by changing the venom composition of the wasp when injecting eggs, allowing escape from the encapsulation immune response of the host. Inverse effects were reported where high temperatures (over 35 °C) affected the growth and development of the parasitoid wasp *Campoletis chlorideae* Uchida, 1952 in its host, the cotton bollworm *Helicoverpa armigera* (Hübner, 1808) [85]. However, in this case, *C. chlorideae* parasitism is utilized for crop pest control [86], and the negative effects of temperature on its survivability and development in its host would prove drastic and give an advantage to the cotton bollworm pest.

Concerns also rise in the subject of disease-transmitting insects like the mosquito *Aedes aegypti* Linnaeus, 1762, showing that there is a variation in thermal tolerance between mosquito populations, stating that thermal performance insect models may be unsuitable for assessment considering climate change [87]. Furthermore, the status of climate change will result in decreased insect generation time and increase the diseases transmitted via insects [88].

### 6.2. Impacts on Beneficial Insects

Beneficial insects such as honey bees and fig wasps are especially sensitive to climate-driven stress. While moderate warming may support colony productivity in some regions [89,90], high temperatures have been shown to increase mortality, oxidative stress, and water loss during overwintering phases [91]. Notably, differences in thermal tolerance between species such as *A. cerana* versus *A. mellifera* highlight uneven vulnerabilities. These ecological impacts are compounded by physiological trade-offs involving immune suppression and microbiome disruption under heat stress, as described in Section 4.2. Together, these stressors reduce pollination efficiency and increase colony collapse risk, particularly under combined pressures from pathogens and pesticides.

On the other hand, data modeling suggests that increasing temperatures will lead to increased invasion by the yellow-legged hornet *Vespa velutina nigrithorax,* especially in Central and Eastern Europe [92], further risking bee colonies. However, the implication of temperatures in species invasion must be further evaluated for more concrete examples. In addition, wasps such as *Polistes biglumis* (Linnaeus, 1758) and *Polistes gallicus* (Linnaeus, 1767) exhibit behavioral adaptations to temperature changes, including cooling their brood and/or adjusting their nesting preferences, which enable higher temperature tolerance [93]. Thus, the behavior of the species is also a contributing factor that determines the general favoritism/disfavor of insect species compared to each other. In contrast, pollinator wasps of fig trees are negatively affected by increasing temperatures despite their small size [94]. Indeed, the interaction between insects and the environment cannot be truly illustrated by single parameters.

**Figure 2:**
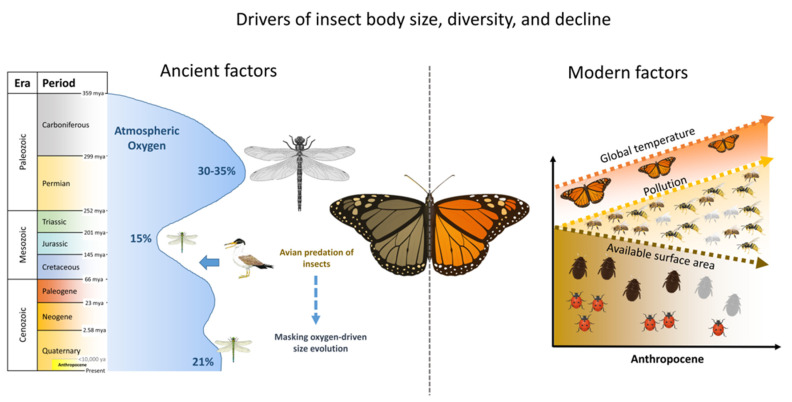
Conceptual comparison between ancient environmental drivers and modern anthropogenic stressors impacting insect evolution and survival. The left image illustrates the ancient drivers that dictated insect populations from the Carboniferous period, 350 million years ago (mya) until the present time. The right image illustrates the modern factors in the Anthropocene that affect insect populations, showing parallelism with ancient effectors.

In Figure 2, we illustrate the simplified comparison between ancient and modern factors of insect population evolution, where the eras and periods of onset were set according to The ICS International Chronostratigraphic Chart (excluding the Anthropocene, which was adapted to the figure by the authors) [95]. The main prehistoric driver affecting insect physiology was the atmospheric oxygen level (Figure 2, left), where the insect body size increased with increasing atmospheric oxygen, but the association was disrupted by the composition posed by ancient birds that preyed on large insects, resulting in decoupling of the effect of oxygen on insect size.

However, some aerial insectivorous birds, such as swifts and swallows, have not declined uniformly [96,97]. In fact, in certain regions, they appear to be expanding into new or urbanized areas. These species feed on aeroplankton, a diverse assemblage of airborne small insects and spiders transported by air currents (e.g., ballooning arachnids) [98]. While overall insect biomass may be decreasing, the spatial and seasonal dynamics of aeroplankton remain underexplored, and could provide intermittent or alternative prey sources for some vertebrates. Such complexity highlights the need to avoid oversimplified assumptions about insect-linked trophic collapse.

The Anthropocene indicated in Figure 2 is the epoch in which human activity has begun to significantly affect the environment. The exact starting point of this epoch is still debated, as there are conflicting definitions. As a result, the Anthropocene is not yet officially recognized as a geological epoch, but the term is widely used across the scientific literature and humanitarian studies [99]. The debate concerns the starting point of the Anthropocene from early agriculture to the start of fossil fuel consumption or the great acceleration in the 1950s. However, in this paper, we will consider the appearance of agriculture (about 10,000 years ago) as the onset of the Anthropocene [99,100].

Generally, this epoch is characterized by the profound impact of human activity on the environment, ultimately leading to global warming, habitat loss and fragmentation, and limited space due to the exponential growth of the human population. In addition, humans continue to contaminate the environment. Altogether, human activities have had a significant impact on wildlife, particularly insects, affecting their abundance, population diversity, body size, and the interactions between pest and beneficial species (Figure 2, right).

### 6.3. Modern Approaches in Measuring Insect Decline and Trait-Based Population Shifts

Accurate assessment of insect decline requires robust, standardized, and scalable monitoring methods. Traditional approaches relying on biomass traps and long-term abundance surveys have been valuable, yet they often lack taxonomic resolution and geographic coverage. Recent advances in molecular tools, remote sensing, and ecological modelling now offer more comprehensive insights into insect diversity dynamics (Table 1).

Trait-based ecological modelling has also become central to predicting insect abundance and responses to environmental change [101]. By incorporating traits such as body size, dispersal ability, or reproductive strategy into models, researchers can forecast shifts in species distributions and ecosystem functions. These models, when paired with spatial environmental datasets such as land use and/or temperature anomalies, can help identify both vulnerable taxa and emerging pest risks. Long-term ecological monitoring networks such as the German Malaise Trap provide essential temporal data but are limited in taxonomic depth as it mainly limited to flying insects [102].

Emerging technologies, such as DNA metabarcoding of bulk samples or environmental DNA (eDNA) mainly from soil and water, enable rapid and high-resolution identification of insect taxa, including cryptic or morphologically indistinct species [103]. These molecular approaches expand the detection range and reduce reliance on expert taxonomists, though their integration into standardized protocols remains ongoing. There are several beneficial applications for these technologies in environmental studies. For example, sampling eDNA allows the detection of rare and/or invasive species, monitoring of population dynamics, assessment of anthropogenic impacts, and other environment-related assessments [104]. Though aquatic environments still dominate sampling efforts, studies are increasingly expanding into other ecological niches and enabling broader taxonomic detection [105]. Despite limitations, the advancement in technologies has broadened the possibilities and capacity for biodiversity assessment. Drones can be integrated with eDNA collection from plant surfaces as a reliable and advanced method while overcoming the limitations of soil sampling, which gives localized information, or the weak detection of eDNA from air samples [106]. The analysis of eDNA has been effectively applied to assess biodiversity, identifying that 83.5% of individually sequenced species of arthropods and metabarcoding of bulk insect samples from Malaise traps has demonstrated high-throughput potential for monitoring population-level changes in insect communities [107] in addition to rare or cryptic insect species across a variety of ecosystems, enhancing biodiversity assessments and guiding conservation priorities [108,109]. However, while emerging tools such as eDNA, remote sensing, and AI-based models offer substantial promise for insect monitoring, their effectiveness remains dependent on robust taxonomic frameworks. With only around 20% of insect species formally described [110], the expertise of traditional taxonomists is indispensable. These tools should be viewed not as replacements but as complementary assets that can accelerate, augment, and help prioritize taxonomic work, especially in cryptic or understudied taxa.

In addition to the mentioned techniques, remote-sensing technologies can be used to monitor real-time insect trends according to characteristics such as frequency and harmonics of wing flaps and/or light backscattering [111]. Empirical examples can be viewed in a review by [111] mentioning wing beat frequency monitoring, which allowed the accurate determination of 79% of 10 mosquito species and 99.4% of their gender. Remote sensing can also be automated, allowing the continuous retrieval and analysis of collected data [112]. Rydhmer et al. (2022) have developed sensors able to monitor insects automatically without supervision [112].

The potential of the mentioned approaches can be brought on by integration with artificial intelligence (AI) technologies. With its rapid advancement, AI is becoming increasingly integrated into many domains including ecology [113] and could prove to be a valuable tool in biodiversity studies. It has the potential to assess and forecast changes in insect populations, as well as to perform image recognition and AI-powered species identification. These applications can reduce the time needed for analyses and may also minimize human error and biased assumptions. Such an integrated monitoring strategy is essential for informing conservation policies and evaluating the effectiveness of mitigation efforts.

However, while eDNA and AI-based tools hold great promise for improving insect monitoring, they are not substitutes for traditional taxonomic expertise. With only ~20% of insect species formally described (Li & Wiens, 2023) [110], integrative approaches remain essential for robust biodiversity assessment.

## 7. Discussion

Several parameters are needed to assess the underlying causes of insect decline in a more comprehensive approach beyond toxicological studies of the effect of pollutants. These global studies would also make it possible to establish correlations between the insect decline linked to (1) exposure to pesticides; (2) variations in the composition of the atmosphere (oxygen) and other atmospheric parameters (density, climate change); and (3) insect density, size, distribution, total biomass, and diversity over time. This will facilitate understanding the level of contribution of different factors to insect decline and the evolutionary selectivity of insects depending on atmospheric and environmental conditions regarding climate change and available space for expansion and reproduction. The combined effect of environmental factors and anthropogenic activity decreases insect fitness despite assessments of single exposures to limited parameters.

In a sense, pesticide application with its indirect effect on insects is like rapid environmental changes when insect species are unable to adapt as rapidly or when the favor shifts towards pesticide resistance in pests. Pesticide resistance is observed in pests such as ticks [114,115], aphids [116], and moths, as seen in the case of pesticide resistance in the diamondback moth in the context of climate warming [117]. In addition, pesticide resistance was also seen globally in other species of moths, thrips, beetles, mosquitoes, and cockroaches [118]. Thus, pesticide application can have drastic effects by generally affecting insect immunity, inducing resistance in pest insects, and disrupting the ecological balance.

Climate change is indeed alarming despite any counterarguments as the relatively rapid change in temperature and oxygen levels has resulted in evolutionary selection and species decrease even before human civilization. Moreover, insects are diverse and are affected differently by climate change and anthropogenic activity, leading to variable declines within insect orders and species in general. This may disrupt the balance of the ecosystem not just in the realm of insects but also in the whole biosphere due to the crucial role of insects. Nowadays, anthropogenic activity is altering the biosphere and causing massive changes in species distribution and selection. In a way, humans control the environment and therefore control the fate of species, especially insects [119].

Long-term studies on the effect of climate change on insects are geographically and taxonomically narrow [120]. Thus, more long-term and comprehensive studies are needed to view the true extent of insect decline. In addition, the variation between results comprising the effect of temperature on insect immunity, survival, its relation to body size, and interaction with diseases requires comprehensive models to allow a more accurate prediction of insect decline and selective pressure attributed to climate change and anthropogenic activity.

Trait-based risk modeling frameworks increasingly incorporate physiological thresholds and life-history traits, offering predictive power that aligns with evolutionary legacies [101]. Integrating palaeobiological insights such as past insect responses to oxygen and temperature shifts could refine current environmental risk assessments, making them more evolution informed [24]. These advanced tools can enhance our ability to detect environmental changes, track population dynamics, and predict ecosystem responses. However, to ensure robust and reliable outcomes, these innovative methods should be complemented by comprehensive long-term monitoring and consistent data collection efforts. Integrating cutting-edge technologies with traditional ecological monitoring will provide a more holistic understanding of insect diversity trends and help inform effective conservation strategies.

## 8. Conclusions

Understanding the parallels between ancient environmental shifts and modern anthropogenic impacts is essential for predicting and mitigating the rapid loss of insect diversity in the Anthropocene. The comparison between ancient environmental drivers and modern anthropogenic factors offer crucial insights for understanding evolutionary legacy inducing inherited physiological limits as responses to stressors, revealing mechanistic similarities, and highlighting evolutionary constraints. As a result, we will be able to attain better predictive power in modeling and anticipating the effect on modern stressors on insects, increasing the robustness of risk assessment. Typically, environmental risk assessments treat modern stressors in isolation. However, viewing them as an intensification of ancient pressures calls for more holistic frameworks that integrate evolutionary history with current exposures.

Typically, such assessments treat stressors in isolation. However, recognizing modern stressors as intensified versions of ancient selective forces calls for more holistic frameworks that integrate evolutionary history with present-day exposures. Recent long-term studies report over a 75% decline in flying insect biomass across protected areas over 27 years [121], a rate unmatched even during major turnover events seen in the insect fossil record [39].

The dramatic findings of insect decline were widely publicized [122] and played a central role in drawing scientific and public attention to the insect biodiversity crisis. However, as Kunin (2019) points out, such studies face methodological limitations including opportunistic sampling and lack of species-level data, constraining their generalizability [123]. These issues underline the need for coordinated standardized insect monitoring frameworks globally.

Comparative trends suggest that the pace of modern insect diversity loss likely exceeds that of historic evolutionary bottlenecks, highlighting the severity of current anthropogenic pressures [2].

To move forward, we recommend implementing long-term, evolution-informed monitoring strategies that leverage emerging technologies such as eDNA, remote sensing, and AI-powered trait modeling. These tools should be embedded within standardized protocols across ecosystems and extended over broad temporal and spatial scales. This approach will enable more reliable detection of trends in insect diversity, support early identification of at-risk taxa, and ultimately inform more effective conservation actions. By aligning modern monitoring efforts with deep evolutionary context, a more predictive and resilient foundation for insect diversity assessment and protection can be built.

## Figures and Tables

**Table 1:** Modern approaches in monitoring and predicting insect population dynamics.

Approach	Description	Strengths	Key Indicators
Long-term trait-based tracking and modelling	Monitors ecological functions such as pollination, decomposition, predation, or other functions, and physiological traits to predict vulnerability or adaptability	Forecasts responses to environmental stressors. Links biodiversity to ecosystem function. Standardized detection of insect trends over decades.	Trait distribution, functional diversity Shift in insect population dominance, biomass, and functional dominance.
Environmental DNA analysis and metabarcoding	Detection of insect DNA from environmental matrices (mainly plant surfaces followed by soil and air). High-throughput sequencing of mixed insect or environmental samples.	Non-invasive, high taxonomic resolution; detects cryptic species.	Presence/absence, richness, detection probability, taxonomic diversity indices.
Remote sensing	Spatial mapping of habitat changes, fragmentation, or land-use intensity.	Broad spatial coverage, real-time monitoring, links habitat dynamics to insect trends.	Sound frequency, wing beat harmonics, melanization, and flight orientation, automated.
Integrating assessments with artificial intelligence	Generating forecasts in population dynamics. Identification of species.	Bypasses tedious work loads, increases feasibility of wide range assessments. Expanding applications. Limited human bias.	Traits and characteristics, species detection/absence.

## Data Availability

No new data were created or analyzed in this study. Data sharing is not applicable to this article.
